# 3D nanomolding and fluid mixing in micromixers with micro-patterned microchannel walls

**DOI:** 10.1186/s40580-017-0098-x

**Published:** 2017-03-01

**Authors:** Bahador Farshchian, Alborz Amirsadeghi, Junseo Choi, Daniel S. Park, Namwon Kim, Sunggook Park

**Affiliations:** 1 Mechanical and Industrial Engineering Department and Center for Bio-Modular Multiscale Systems for Precision Medicine, Louisiana State University, Baton Rouge, LA 70803 USA; 2 School of Engineering, Texas State University, 601 University Drive, San Marcos, TX 78666 USA

**Keywords:** 3D molding, Surface structures in microchannel, Secondary flow, Advection, Flow patterns

## Abstract

Microfluidic devices where the microchannel walls were decorated with micro and nanostructures were fabricated using 3D nanomolding. Using 3D molded microfluidic devices with microchannel walls decorated with microscale gratings, the fluid mixing behavior was investigated through experiments and numerical simulation. The use of microscale gratings in the micromixer was predicated by the fact that large obstacles in a microchannel enhances the mixing performance. Slanted ratchet gratings on the channel walls resulted in a helical flow along the microchannel, thus increasing the interfacial area between fluids and cutting down the diffusion length. Increasing the number of walls decorated with continuous ratchet gratings intensified the strength of the helical flow, enhancing mixing further. When ratchet gratings on the surface of the top cover plate were aligned in a direction to break the continuity of gratings from the other three walls, a stack of two helical flows was formed one above each other. This work concludes that the 3D nanomolding process can be a cost-effective tool for scaling-up the fabrication of microfluidic mixers with improved mixing efficiencies.

**Graphical abstract:**
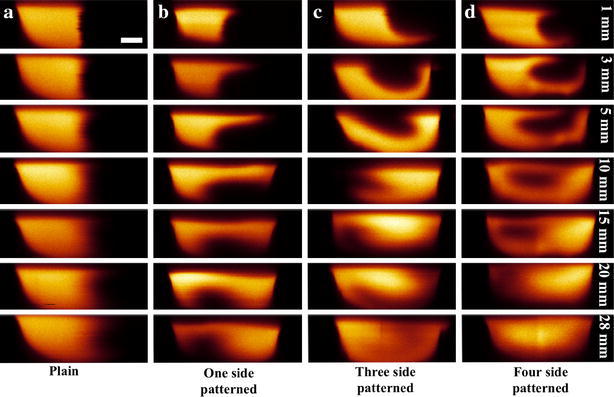
In this paper we show that a micromixer with patterned walls can be fabricated using 3D nanomolding and solvent-assisted bonding to manipulate the flow patterns to improve mixing.

In this paper we show that a micromixer with patterned walls can be fabricated using 3D nanomolding and solvent-assisted bonding to manipulate the flow patterns to improve mixing.

## Background

Over the past two decades microfluidic devices have received significant interests for analytical chemistry, clinical diagnosis, environmental monitoring and food analysis applications [1–7]. Microfluidics offers a variety of advantages such as low consumption of sample and reagents, low power consumption, reduced cost, high throughput, integration of various functionality and automation [8]. Despite the progresses made in recent years, mixing in microfluidics still remains as a challenge, mostly arising from a low Reynolds number (Re) by the small dimensions of microchannels and the limited range of obtainable velocities in its operation. Therefore, the flow regime is mostly laminar and, consequently, in fluidic channels with smooth walls and no external disturbance source the only mechanism through which mixing can occur is diffusion, which is an inherently slow process. The requirement of a long channel for complete mixing increases the device footprint significantly, making the device impractical for many lab-on-a-chip applications. In order to overcome the limitation, tremendous efforts have been given on micromixers, as summarized in the following.

Micromixers can be classified as passive and active micromixers [9]. Detailed information on the design and operating principles of active and passive micromixers can be found in previous reviews [9–12]. In active micromixers where disturbance in fluid flow is generated by an external source such as magnetic field, electric field, ultrasonic effects and thermal medium, their integration into microfluidic devices is challenging due to expensive and complex fabrication protocols and supporting external equipment.

Passive micromixers that do not require an external source can be categorized further into two types: geometrical modification in microfluidic channel design [13–17] and modification of mixing channels by integration with additional structures [18–23]. The geometrical modification for the first type include split and recombination of microchannels [13], hydraulic focusing by using sheath flows provided through two other inlets [14], a modified Tesla structure that induces the Coanda effect to improve mixing [24], curved microchannels [16, 17] and 3D serpentine channels with recurring “C-shaped” units [15]. Their mixing is enhanced either by lamination of fluid streams or by inducing chaotic advection at corners. While their fabrication mostly involves design of a photomask followed by a single step photolithography, it is usually accompanied by an increase in the device footprint, which is not desired in many applications.

In the second group of passive mixers, the additional structures integrated into mixing microchannels can be obstacles, surface patterns at the channel walls or the combination thereof [18–23]. This type of micromixers are interesting particularly because they are effective even for low Re flows [18]. In a phenomenal work by Stroock et al. [20], staggered herringbone structures and slanted well structures were formed on the bottom of the mixing channel using a two-step photolithography and PDMS casting. In Johnson et al. [19], a preformed T-micromixer imprinted in polycarbonate was post-modified with a pulsed UV excimer laser to form slanted wells at the junction. However, because the laser milling is a serial fabrication method, only a small portion of the micromixer channel could be modified with slanted wells. After initial work by Stroock et al. and Johnson et al., researchers added structures more than one surface of mixing microchannels to further improve mixing. In Yang et al. [22], a connected groove micromixer whose bottom and side walls were patterned with connected grooves was fabricated via a two-step photolithography and PDMS casting. Sato et al. [23] demonstrated a 3D microchannel whose top and side walls were patterned with slanted microgrooves. However, their fabrication involves two inclined backside photolithography steps and two topside photography steps.

The literatures indicate that mixing in microchannels can be further improved if a more number of walls are decorated with additional microscale structures. However, the integration of additional structures on the walls of microchannels requires a number of additional micromachining steps or use of high-end equipment and thus increases the fabrication cost significantly [19–23]. Therefore, fabrication of such structures at low cost and with high throughput is a huge technological challenge.

Previously, we have developed 3D nanomolding, a modified molding technique allowing for fabricating micro/nanostructures along the surface of any arbitrary microstructures in polymer substrates with the help of a thin intermediate polydimethysiloxane (PDMS) stamp introduced between the brass mold insert and polymer substrate for molding [25, 26]. The objectives of this paper are two-folds. The first objective is to demonstrate the feasibility of the 3D nanomolding process for simple production of microfluidic devices with fluidic walls decorated with micro- and nanoscale patterns. The other objective is to systematically investigate the improvement of mixing as different numbers of the microchannel walls are decorated with microgratings. Such a systematic investigation has been difficult to achieve due to the lack of a simple and low cost fabrication method for sidewall patterns. Using the 3D nanomolding process, continuous microscale ratchet gratings were patterned on the sidewalls and bottom of the microchannel to realize 3D T-micromixer structures. By using a solvent-assisted bonding technique with a plain or micropatterned cover plate, enclosed T-micromixers with no side, one side, three sides and four sides patterned with microscale ratchet gratings were fabricated and 3D flow patterns induced by the surface structures at different locations of the microchannels were studied using confocal microscopy.

## Methods

### Brass mold fabrication and PMMA pre-patterning

Two different molds were used for pre-patterning of micro/nanostructures in poly(methyl methacrylate) (PMMA) substrate. A Si stamp with an array of nanoholes of 100 nm diameter, 200 nm period, and 100 nm height was used for nanopatterning and a brass mold containing ratchet gratings with the period of 75 μm was used for micropatterning. Another brass mold with a T-junction protrusion was used produce a micromixer, The width and depth of the microchannels for the T-junction micromixer were 50 and 70 μm, respectively. The length of the fluid inlet to the T-junction was 5 mm and the length from the T-junction to the fluidic outlet was 30 mm. The brass molds were fabricated by a KERN MMP2522 micro milling machine. The brass was rough cut with an 800 μm diameter end mill (PMT Tools) at 200 mm/min, followed by a finishing pass with a 100 μm diameter end mill (PMT Tools) at 75 mm/min. The spindle was run at 40,000 rpm for all passes. For ratchet fabrication, a jig was used to angle the brass surface off the horizontal. After fabrication, the Si or brass mold with nanoholes or ratchet gratings was imprinted into poly(methyl methacrylate) (PMMA) using a commercial nanoimprint lithography (NIL) machine (Obducat 6 inch NIL). Imprinting was performed at 160 °C and 30 bar for 10 min. Then the system was cooled down to 70 °C and demolding was done.

### 3D nanomolding

**Fig. 1:**
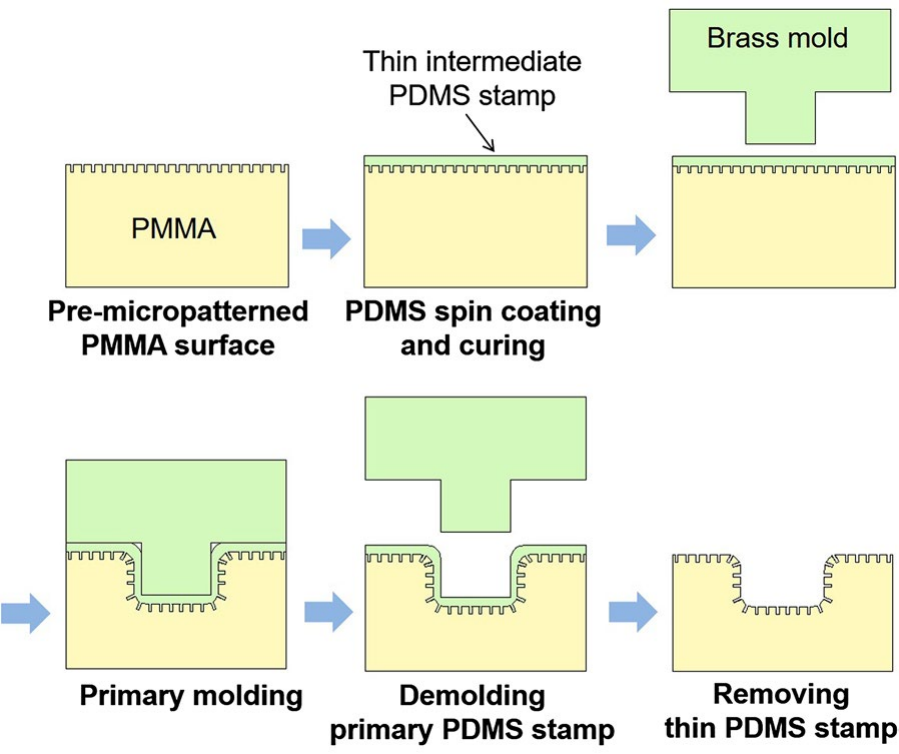
Schematic illustartion of the 3D nanomolding process [26]

Figure 1 shows the process scheme for 3D nanomolding [26]. On the surface of PMMA substrate pre-patterned with nanopillars or micro ratchet gratings, PDMS prepolymer was spin coated at 2000 rpm for 40 s and cured in order to form a thin intermediate PDMS stamp. The thickness of the intermediate PDMS stamp was 41.6 ± 2.5 μm. This was followed by the primary molding step at 170 °C and 5 bar for 5 min, which was performed in the NIL machine using a brass mold having microfluidic protrusion structures. For microfluidic devices for mixing experiments, the angle between the direction of ratchet gratings in the pre-patterned PMMA substrate and the direction of the microchannel in the brass mold was set to be ~45°, so that slanted ratchet gratings were formed on the sidewalls and bottom surface of the microchannel. After primary molding, demolding was performed in two steps at 70 °C: first demolding of the brass mold and then second peeling off the PDMS intermediate stamp from the 3D molded PMMA substrate. Even for fabricating plain microchannels, a thin intermediate PDMS stamp without any pre-patterned structures was used for primary molding in order to obtain a similar cross-section to those for 3D microchannels decorated with ratchet gratings.

### Solvent-assisted bonding

Before bonding a cover plate to 3D molded substrate, holes were drilled in the inlet and outlet area of the 3D channel. A solvent-assisted bonding technique developed by Brown et al. [27] for bonding of PMMA chips was used. A few drops of a solvent mixture (47.5% dimethyl sulfoxide (DMSO), 47.5% water and 5% methanol) were spread over the cover plate and then the 3D molded substrate was placed in conformal contact with the cover plate. The assembly of 3D molded substrate/cover plate was loaded in the NIL machine and brought to 92 °C and a 10 bar for 30 min, which led to complete bonding. For micromixers with no side and three sides of microchannel walls patterned, a plain cover plate without any patterns was used while, for micromixers with one side and four sides of channel walls patterned, a cover plate patterned with ratchet gratings was used. For the micromixer with four sides patterned, the cover plate was so aligned for bonding that the ratchet gratings in the cover plate were in parallel with the ratchet gratings in the bottom of the 3D microchannel.

### Leakage testing

After solvent-assisted bonding, PEEK™ tubing capillaries (part number 1577-12x, IDEX, Oak Harbor, WA) with 795 μm (0.0313 in) outer diameter and 177 μm (0.007 in) inner diameter were inserted into inlet/outlet ports of the chip and a tiny amount of epoxy resin was applied to the capillary-chip interfaces to prevent leakage. Two 5 mL glass syringes (1005TLL, Hamilton, Reno, NV, USA) were filled with a fluorescein dye solution (fluorescein sodium salt in deionized (DI) water with a concentration of 3.75 × 10^−2^ g/L, F6377-100G, Sigma-Aldrich, St. Louis, MO, USA). The dye solution was injected at 40 μL/min into the inlets of the micromixer using syringe pumps (KDS220 multi-syringe pump, Kd Scientific Inc., MA, USA). The injected micromixer was imaged using an inverted fluorescence microscope (Eclipse, Nikon Instruments Inc. Melville, NY, USA) with a 10× objective lens for leak test and the images were captured by a digital camera (Cool- SnapFX Photometrics, Tucson, AZ, USA).

### Mixing characterization

A scanning laser confocal microscope (Leica TCS SP2) was used to map out 3D flow patterns induced by the surface structures on microchannel walls at different locations along the microchannel (1, 3, 5, 10, 15, 20 and 28 mm) from T-junction. DI water and a solution of fluorescein dye in DI water were injected from separate inlets of the T-micromixer. Two flow rates of 10 and 40 μL/min in the mixing microchannel were investigated. The results from confocal microscopy were a stack of images in the xy plane with a slice thickness of 383 μm. The stack of images was then assembled into a 3D image and transects in the desired area in the xz plane. x, y and z are the coordinates in the direction of the width, length and height of the channels respectively.

To quantify the degree of mixing for a location in 3D microchannels, the standard deviation of the normalized fluorescence intensity, i.e. fluorescence intensity at a location with respect to the maximum fluorescence intensity at the inlet, was obtained from cross-sectional confocal microscopy images using $\sigma = \langle (I - \langle I\rangle )^{2} \rangle^{1/2}$. *σ* is the standard deviation, I is the grayscale intensity value of a pixel with a value between 0 and 1, and 〈 〉 means an average over all pixels in the image. Therefore, *σ* is 0.5 for completely separated fluids and 0 for completely mixed fluids. Only the central 50% of the area of the images was used to calculate *σ* to eliminate variaions of the fluorescence intensity around the walls due to optical effects [20]. *σ* is also called mixing index.

### Numerical simulation of the mixing

**Fig. 2:**
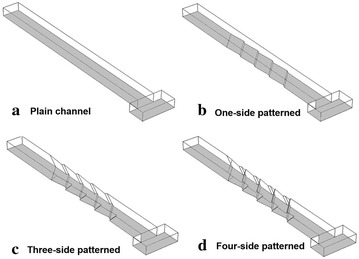
Schematic illustration of **a** the plain **b** one side patterned **c** three sides patterned and **d** four sides patterned micromixers. The ratchets patterned on the surface were aligned at a ~45° angle with respect to the direction of the channel

A series of computational simulations were performed using a multiphysics software COMSOL for four different models shown in Fig. 2. The models consisted of a T-micromixer with a 4000 μm long microchannel with a cross-section of 60 μm wide and 30 μm height. The four models differed by the number of microchannel walls decorated with microscale ratchet gratings. The high and period of the ratchets were 6 and 75 μm, respectively. The ratchets were placed at a 45° angle to the long axis of the channel.

The models used for the simulation were different from the fabricated structures used for mixing experiments in three aspects. First, the dimensions of the T-micromixer channels were reduced by a factor of ~2 with respect to those of the micromixers used for experiments. This means that the cross-sectional area of the long microchannel was reduced by a factor of ~4. The height of the ratchet gratings was also increased by a factor 1.5. These dimensions were used to achieve simulation results in a reasonable computational time. Second, ratchet gratings in the models were formed on the walls of a portion of the long microchannel. Starting at a 150 μm distance from the T-junction of micromixers, only 45 ratchets were placed on the walls of microchannels. On the other hand, in micromixers used for experiments, entire microchannel walls were decorated with ratchet gratings. Third, the cross-section of the model microchannel was rectangular while that for 3D microchannels had rounded bottom corners which were inevitably produced during the 3D molding process. The previous systematic study on 3D molding indicates that the roundness of corners in 3D microchannels can be reduced by using a thinner intermediate PDMS stamp and by changing dimensions of microchannels [25, 28].

Two liquids with properties of water at 298 K were let through each inlet of the micromixer. One of the liquids contained 1 mol/m^3^ of an imaginary species with an arbitrary diffusion coefficient of 10^−10^ m^2^/s while the other was pure water. The inlet boundary conditions were set at a constant velocity (0.9 cm/s). No-slip boundary condition was used for the walls. The outlet boundary condition was set at zero pressure. The geometry was discretized using the physics-controlled mesh module of the software. A multi-physics finite element model for an incompressible steady state flow consisting of the Navier Stokes equation in the laminar regime and the convective diffusion was solved by Comsol at each node. Mixing was quantified by comparing the standard deviation of the concentration profiles.

## Results and discussion

### Structural characterization of fabricated devices

**Fig. 3:**
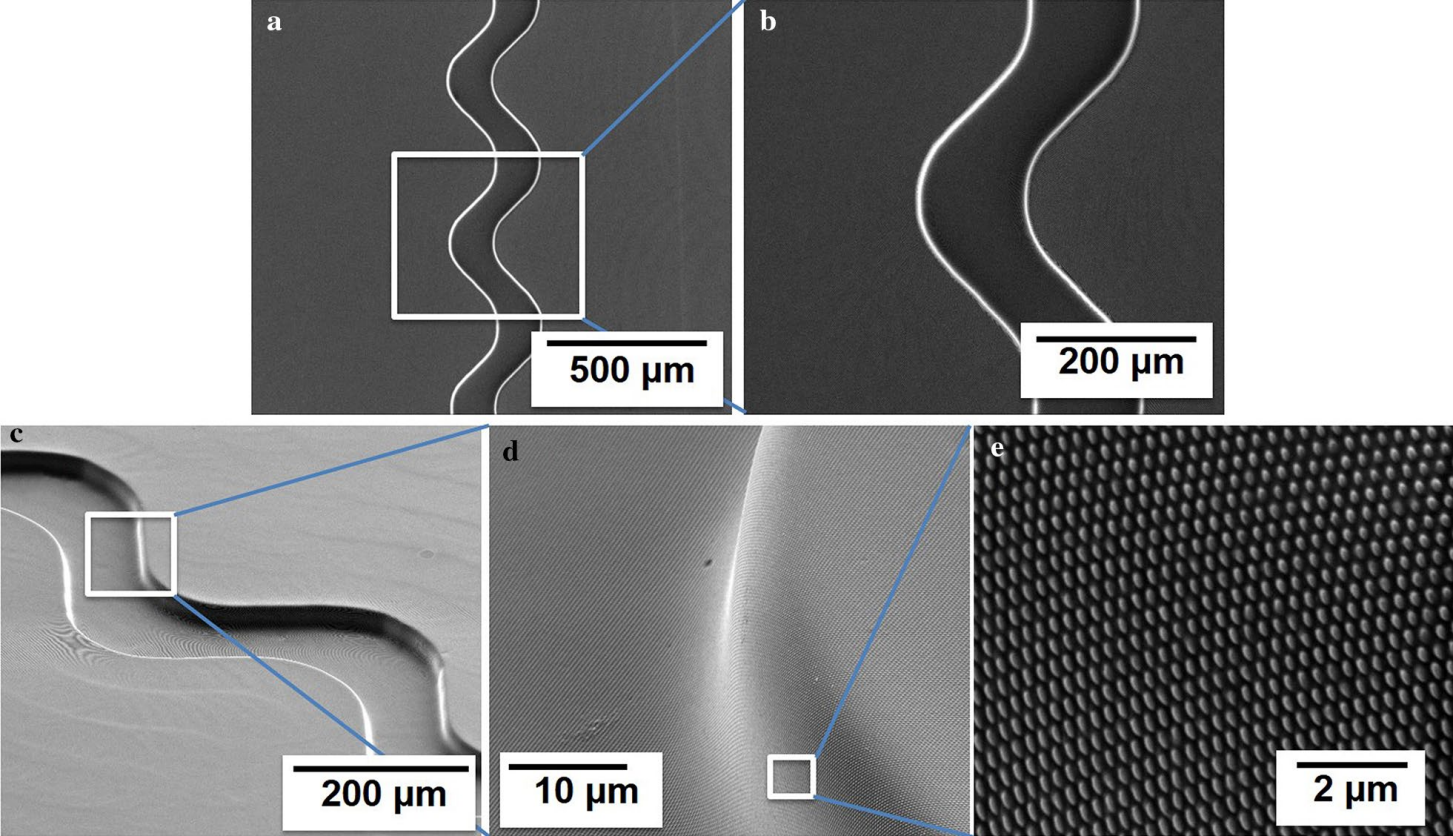
SEM micrographs of a 3D nanomolded sinusoidal microfluidic device in PMMA. **a** and **b** are top view images with different magnifications and **c**, **d**, and **e** correspond to images with a tilted angle with different magnifications

Figure 3 shows scanning electron microscope (SEM) images for a 3D nanomolded PMMA substrate in which a sinusoidal microfluidic channel was decorated with nanopillars. The corners of the microchannel were slightly rounded, which was inevitably produced during the 3D nanomolding process. However, nanopillars were well formed on the entire surface of the microfluidic channel, which indicates that 3D nanomolding is a feasible method to produce nanostructures on the walls of microfluidics.

**Fig. 4:**
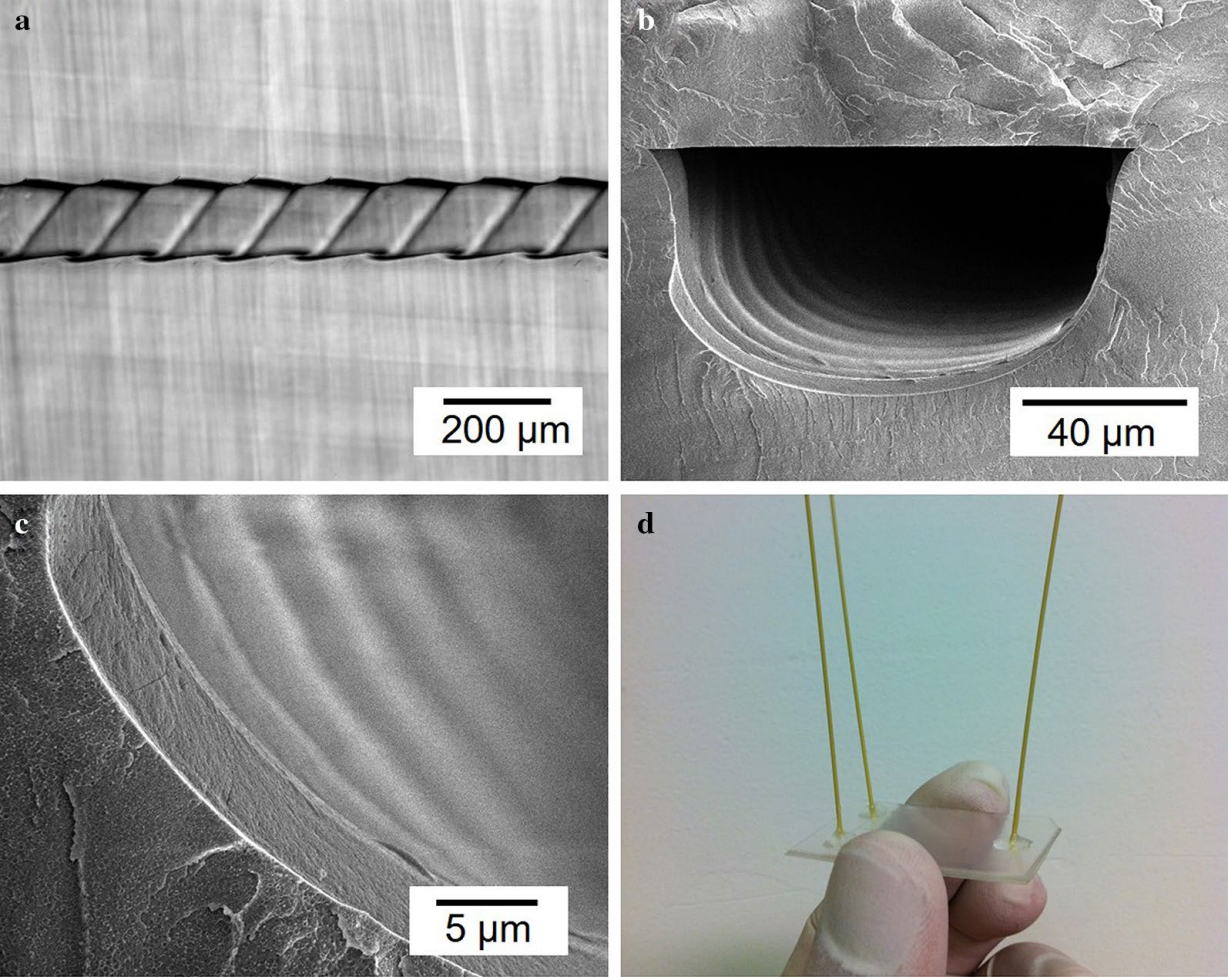
SEM micrographs of **a** a 3D channel fabricated via 3D molding, **b** the cross-section of the 3D channel after solvent-assisted bonding, **c** micro ratchets integrated inside the channel. **d** A photograph of the micromixer with patterned walls

Figure 4a shows SEM images of a 3D molded PMMA substrate in which ratchet microgratings were formed on the entire surface of the substrate. The 3D microchannel had the depth and width of 65.0 ± 2.0 μm and 129.5 ± 4.9 μm, respectively. The ratchet structures in the bottom center of the microchannel had the height of 3.5 ± 0.2 μm and were aligned at a ~45° angle with respect to the direction of the microchannel.

It is challenging to form an enclosed fluidic chip via bonding a cover plate to the 3D molded substrate due to the presence of microscale ratchet gratings on the top surface of the substrate. For this purpose, solvent-assisted bonding is preferred to thermal bonding which is the bonding method for most polymer microfluidic applications. In thermal bonding, an elevated temperature close to the PMMA glass transition temperature (T_g_) of 105 °C is used [27]. At this temperature, PMMA chains are mobile and thus deformation of the molded microstructures can occur. In solvent-assisted bonding, on the other hand, a temperature lower than T_g_ of PMMA (85–95 °C) is used. Even though exposure to a solvent enhances softening of PMMA at the surface, only a portion of the exposed PMMA cover plate in contact with 3D molded PMMA substrate can deform during bonding, as demonstrated by Brown et al. [27]. Thus, ratchet gratings formed on the microchannel walls can survive during bonding. Figure 4b, c show the cross-section of a 3D microchannel after bonding to a plain PMMA cover plate. The ratchet gratings on the bottom surface and sidewalls of the microchannel were clearly visible, indicating that the solvent-assisted bonding process did not produce significant deformation on the integrated ratchet structures. The height and width of the 3D channel after solvent-assisted bonding were measured to be 60.5 ± 0.7 μm and 121.5 ± 1.0 μm, respectively, and the height of the integrated ratchet gratings in the bottom center of the channel was 3.7 ± 0.3 μm. Compared to the dimensions of the 3D microchannels prior to bonding, the dimensional variations occurring during the bonding process were <8%. Figure 4d shows a photograph for a complete microfluidic chip with the 3D microchannel after connecting and gluing the capillary tubes to the chip. Leak test results showed no leakage around the 3D microchannel, which in turn confirms that solvent-assisted bonding is a suitable method to form an enclosed fluidic system for 3D molded PMMA substrates.

### Fluid mixing in 3D microchannels

**Fig. 5:**
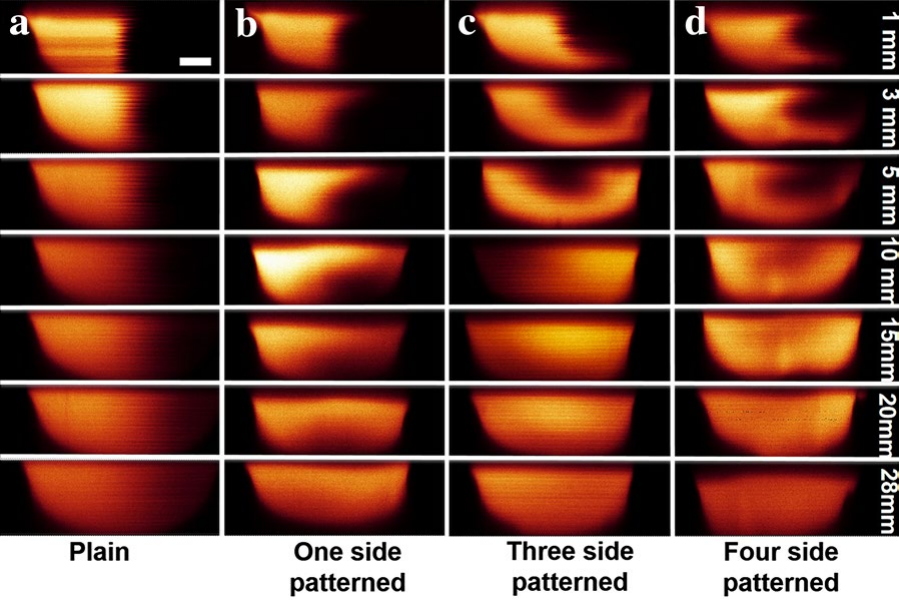
Confocal microscope images for cross-sections of **a** plain microchannel and 3D microchannels with **b** one side patterned (*top side*), **c** three sides patterned (*bottom* and *side walls*), and **d** four sides patterned (*top*, *bottom* and *side walls*) for different locations of 1, 3, 5, 10, 15, 20 and 28 mm from T-junction. Deionized water and a solution of fluorescein dye in deionized water were injected from separate inlets of the micromixer. The volumetric flow rate in the mixing microchannel was 10 μL/min. The *scale bar* is 20 μm

After fabrication, DI water and a solution of a fluorescein dye in DI water were injected from separate inlets of the micromixers and their mixing behavior was studied using confocal microscopy. Figure 5 shows cross-sectional confocal microscopy images taken at different locations of 1, 3, 5, 10, 15, 20 and 28 mm from T-junction for four different mixing microchannels: (1) plain channel and 3D channels with (2) one side patterned (top side), (3) three sides patterned (bottom and side walls) and (4) four sides patterned (top, bottom and side walls). The volumetric flow rate in the mixing microchannel was 10 μL/min which corresponds to a Reynolds number of 1.85. For the plain channel, the dyed water and pure water moved side by side along the channel and thus mixing occurred mainly by diffusion, as can be seen in Fig. 5a. Advection was along the channel and was not useful for transversal mixing.

When one or more sides of microchannel walls were patterned with slanted micro ratchet gratings, the mixing mechanism became a combination of diffusion and advection. Ratchet gratings formed at a ~45° angle relative to the microchannel direction produced a transversal component of advection for fluids adjacent to the ratchet gratings, which in combination with the flow along the channel led to the formation of a helical flow. This can be clearly seen in Fig. 5b–d. The transversal component of flow increased the interfacial area between fluids and cut down the diffusion length for complete mixing. As a result, mixing was improved compared to that for the plain channel.

The helical flow became even more pronounced when three sides of the microchannel walls were patterned with continuous slanted ratchet gratings. Here, “continuous” means that line gratings were formed when two or more consecutive sides of a microchannel were virtually unfolded on a flat surface. Then, the gratings on two opposite sidewalls formed by single 3D molding were perpendicular to each other (Fig. 5c). The enhanced helical flow occurred because continuous ratchets on the left (right) sidewall in Fig. 5c also created transversal downward (upward) flow, which helps the fluids rotate faster as they move along the channel.

For the microchannel with all sidewalls patterned, the ratchet gratings on the top and bottom surface were parallel to each other while those on the two sidewalls were perpendicular to each other. Thus, the continuity of gratings was broken on the top surface. Consequently, an initial stretch of dyed fluid toward the pure water side occurred at both top and bottom surfaces, forming a stack of two helical flows one above the other in opposite directions. The helical flow formed on the bottom of the channel was stronger than the one on the top as it was strengthened by sidewall patterns. In this microchannel, almost complete mixing was achieved at a distance of 10 mm from T-junction.

**Fig. 6:**
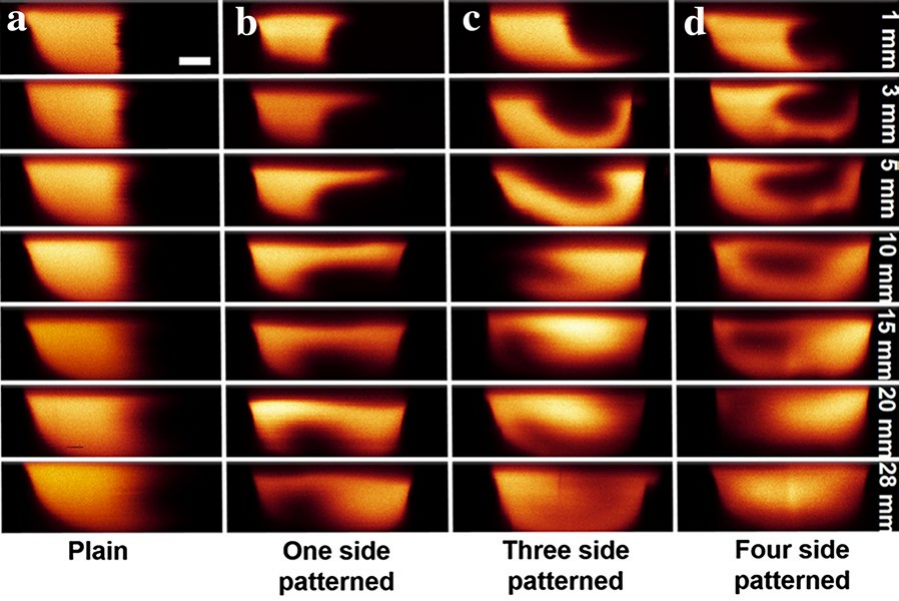
Confocal microscope images for cross-sections of **a** plain microchannel and 3D microchannels with **b** one side patterned (*top side*), **c** three sides patterned (*bottom* and *side walls*), and **d** four sides patterned (*top*, *bottom* and *side walls*) for different locations of 1, 3, 5, 10, 15, 20 and 28 mm from T-junction. Deionized water and a solution of fluorescein dye in deionized water were injected from separate inlets of the micromixer. The volumetric flow rate in the mixing microchannel was 40 μL/min. The *scale bar* is 20 μm

We also studied the mixing behavior at a higher flow rate of 40 μL/min (Re = 7.4) and the fluorescence micrographs are shown in Fig. 6. A similar flow behavior was observed but the degree of mixing was lower compared to the results obtained at a 10 μL/min flow rate.

**Fig. 7:**
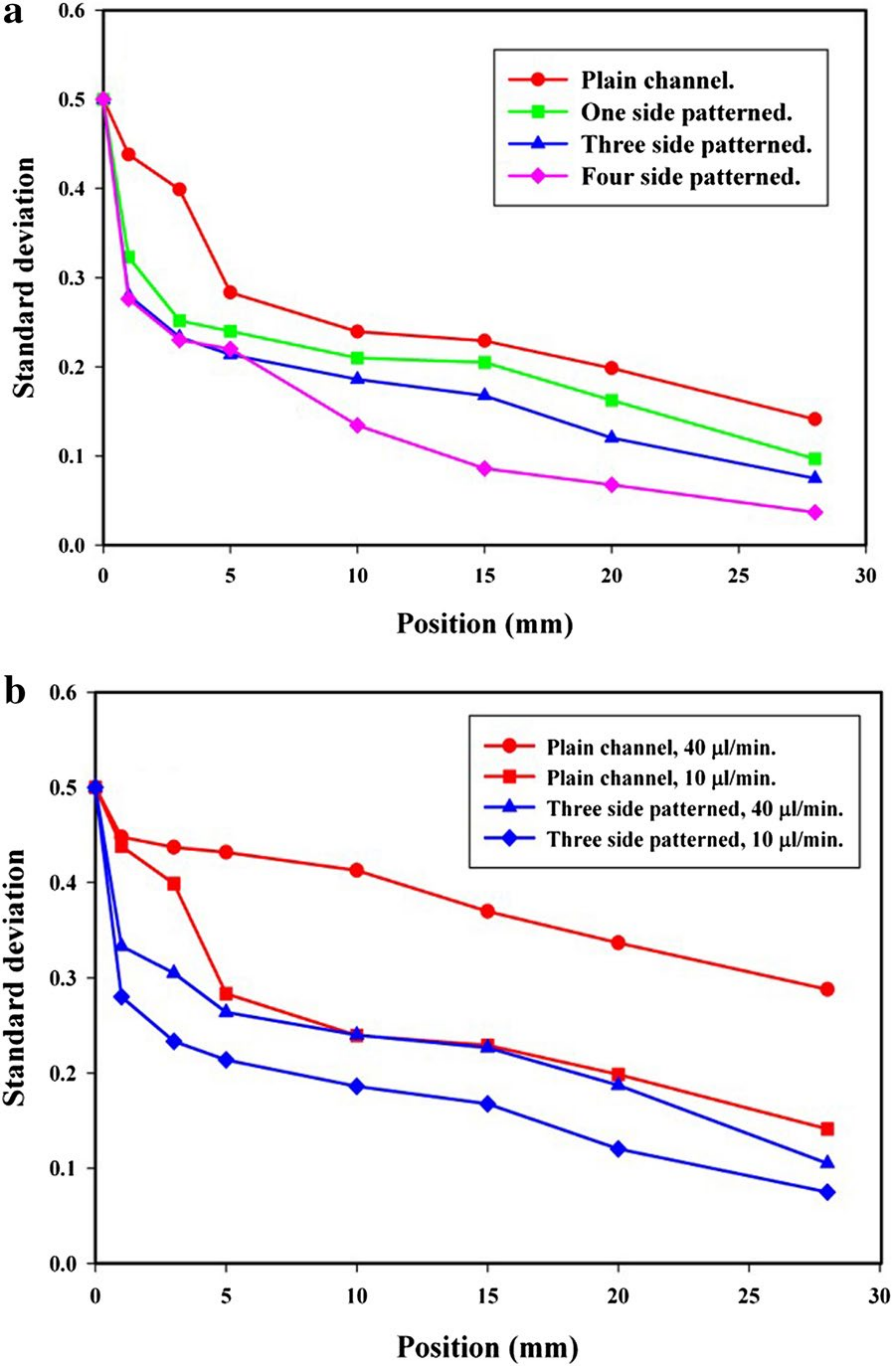
**a** The standard deviation (σ) for the normalized intensity in the cross-sectional confocal microscopy image versus the distance from T-junction for different micromixers (plain microchannel and microchannels with one side, three sides, four sides patterned) at a flow rate of 10 μL/min. **b** σ value versus the distance from T-junction for the plain channel and the 3D channels with three sides patterned at different flow rates of 10 and 40 μL/min

The degree of mixing can be quantified by taking the standard deviation of the normalized intensity from cross-sectional confocal images at different locations in the mixing microchannel. The standard deviation values for different 3D microchannels at 10 μL/min were shown in Fig. 7a. In general, the degree of mixing was in the increasing order of plain microchannel < microchannel with one side patterned < microchannel with three sides patterned < microchannel with four sides patterned.

Figure 7b compares the standard deviation versus location from the T-junction for the plain channel and the 3D channel with three sides patterned at two different flow rates of 10 and 40 μL/min. An increased flow rate for both cases increased the standard deviation (or decreased the degree of mixing) at the same location of the microchannels from T-junction. However, the degree of mixing in the 3D microchannel with three sides patterned at 40 μL/min was still significant and comparable to that in the plain microchannel at 10 μL/min, indicating that 3D microchannels are particularly useful for high flow rate microfluidic applications.

Comparing the position of dyed water front in the microchannel with three sides patterned for two different flow rates (Figs. 5c, 6c), the degree of the spiral rotation induced by the surface ratchet gratings was not much changed by varying the flow rate. At a high flow rate, the time for fluids to reach a location in a microchannel, i.e. the time for diffusion to occur, was short for both plain and 3D channels. However, as a result of the enhanced interfacial area between two fluids by transversal fluid motion in 3D microchannels, the reduction of mixing by an increased flow rate will not be significant compared to that in the plain microchannel.

### Comparison with simulation results

**Fig. 8:**
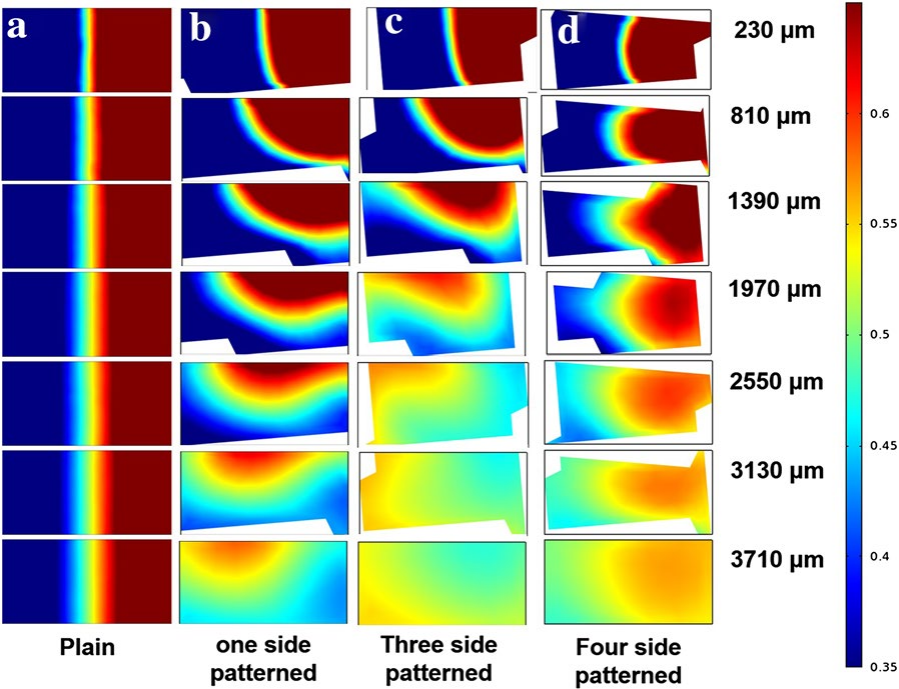
Simulated cross-sectional concentration profiles alongside mixing channels for **a** plain channel and channels with **b** one side, **c** three sides, **d** four sides patterned. A T-micromixer used for the simulation was composed of a 4000 μm long channel with a cross-section of 60 μm wide and 30 μm high. The ratchets with a height of 6 μm and a period of 75 μm were placed at a 45° angle to the long axis of the channel. Flow velocity was 0.9 cm/s

The experimental results were compared with results from numerical simulations. The differences in the models used for simulations from the actual structures used for experiments are described in Sect. 2.6. Despite the differences, numerical simulations provide qualitative comparisons with the corresponding experiments. Figure 8 shows the concentration profile images for mixing of two water-based liquids at different locations along the plain and various 3D microchannels. Qualitatively, the simulation results were in good agreement with the experimental results in that surface ratchet gratings induced transversal motion of fluids. The rotation of fluids was enhanced when more sidewalls were patterned with continuous ratchet gratings (Fig. 8b, c). When ratchet gratings on the top surface were formed in parallel to ratchet gratings on the bottom surface, stretching of fluids occurred at both top and bottom surfaces, in agreement with experimental results (Fig. 8d). However, the degree of stretching on the bottom surface relative to that on the top surface was significantly reduced. This can be seen when the flow patterns for microchannels with four sides patterned (see at 3 mm in Figs. 5d, 6d and at 1390 in Fig. 8d) are compared at the distance showing a similar flow pattern for microchannels with three sides patterned (see at 3 mm in Figs. 5c and 6c and at 1390 µm in Fig. 8c). Thus, in the simulated case, the top helical flow seems to hinder the stretching of fluids on the bottom surface, which will be further discussed in the next.

**Fig. 9:**
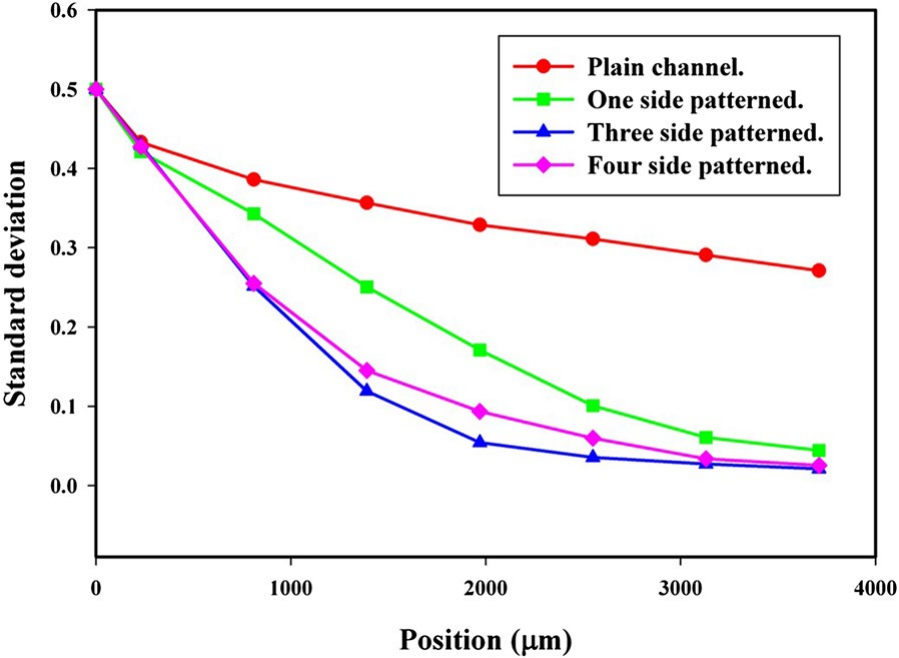
Standard deviation obtained from the simulated cross-sectional concentration profiles shown in Fig. 8

Figure 9 shows the standard deviation of the concentration profiles shown in Fig. 8. Addition of ratchet gratings improved mixing significantly. Due to smaller dimensions of the mixing microchannel, larger size of integrated structures and a slower fluid velocity, mixing occurred in a short length compared to what we observed in the experiments. Mixing was most efficient when the sidewalls and bottom of the microchannel were patterned. However, incorporation of ratchet gratings to the top surface (microchannels with four sides patterned) did not improve mixing further with respect to the microchannel with three sides patterned, which is different from experimental results. We attribute this to the use of a diffusion coefficient value of 10^−10^ m^2^/s for the dyed water, which is lower than ~10^−9^ m^2^/s for a small molecule in water at room temperature [29]. Thus, mixing by diffusion at the interface of stretched liquids seems to be a rate-limiting process over the transverse flow of liquids induced by surface ratchets. In this case, the helical flow formed by the top surface ratchets prevents the interface area of two liquids from further expanding, resulting in a detrimental effect on mixing.

The deformation of microstructures on the surface of microchannel walls during fluidic experiments may be an issue due to a strong shear force applied. We have not performed an SEM investigation after the fluidic experiments. However, an inspection with an optical microscope before and after the fluidic experiments does not indicate any hint that the microstructures at the microchannel walls were deformed. We calculated the shear stress applied of the fluid using a simple Poiseuille model with a parallel plate with infinite aspect ratio in the cross-sectional dimensions where the shear stress is a function of volumetric flow rate, Q, channel dimensions (height h, width w, and length L), and fluid viscosity μ, as follows:

$$\tau = - \frac{12Q\mu }{{h^{2} w}}.$$

Putting the experimental conditions used in this study to the equation (Q = 40 μL/min; μ ~1 Pa s; h = 65 μm; and w = 130 μm) shows a shear stress in the range of ~73,000 Pa. The actual shear stress at the microchannel wall should be even smaller than this value. Even though it is a rough estimation, this value is significantly lower than the tensile stress values of PMMA, which is in the range of 48–76 MPa. Therefore, under the experimental conditions used in this study, it is not expected that the microgratings are deformed during the fluidic experiments.

Finally it should be noted that in most cases microfluidic designs are limited to planar, layer-by-layer geometries that are imposed by current lithography based techniques of microfabrication [20]. Using the 3D molding process, 3D patterns can be imprinted easily in a wide range of thermoplastic polymers used for low cost lab-on-a-chip applications and enclosed microfluidic devices with 3D patterns can be formed via solvent-assisted bonding. The current 3D molding process time is limited by curing of PDMS to form an intermediate stamp. However, the process time can be significantly used by using other UV curable polymers with similar cross-linking densities to have similar elastic properties since UV curing time is much shorter than thermal curing needed for PDMS. Various structures such as hierarchical micro and nanostructures with different geometries and dimensions can be patterned on the walls of microchannels and the cover plate enabling manipulating flow patterns. The direction of the patterns can also be controlled by setting different angle between brass mold protrusions and micropatterns on the surface of the thermoplastic polymer in the modified 3D molding process. Such advantages make the 3D molding process a suitable and powerful technique for fabricating micromixers.

## Conclusions

We studied the effect of the surface structures embedded on microchannel walls in micromixers on the mixing behavior. Four different 3D micromixers with no side, one side, three sides and four sides patterned with microscale ratchet gratings were fabricated via 3D nanomolding and solvent-assisted bonding. In plain channel mixing occurs as a result of diffusion at the interface of fluids which move side by side along the channel. By adding ratchet gratings to the surface of micromixers flow patterns could be manipulated. The strength of the helical flow induced by slanted ratchet gratings was intensified by increasing the number of walls continuously patterned with ratchet gratings. In a micromixer whose all sidewalls were patterned in such a way that ratchet gratings on the top and bottom surface were parallel to each other and those on the two sidewalls were perpendicular to each other, a stack of two helical flows form one above each other, causing one fluid to wrap around other fluid and push it across the 3D channel.
